# Deficiency of the Lysosomal Protein CLN5 Alters Lysosomal Function and Movement

**DOI:** 10.3390/biom11101412

**Published:** 2021-09-27

**Authors:** Indranil Basak, Rachel A. Hansen, Michael E. Ward, Stephanie M. Hughes

**Affiliations:** 1 Brain Health Research Centre and Genetics Otago, Department of Biochemistry, School of Biomedical Sciences, University of Otago, Dunedin 9011, New Zealand; rachelrah@outlook.com; 2 National Institute of Neurological Disorders and Stroke, National Institute of Health, Bethesda, MD 20814, USA; michael.ward4@nih.gov

**Keywords:** Batten disease, iPSC-derived human neurons, CLN5, CRISPR, lysosome function, lysosome acidity, lysosome enzyme activity, cathepsin B, lysosome movement

## Abstract

Batten disease is a devastating, childhood, rare neurodegenerative disease characterised by the rapid deterioration of cognition and movement, leading to death within ten to thirty years of age. One of the thirteen Batten disease forms, *CLN5* Batten disease, is caused by mutations in the *CLN5* gene, leading to motor deficits, mental deterioration, cognitive impairment, visual impairment, and epileptic seizures in children. A characteristic pathology in *CLN5* Batten disease is the defects in lysosomes, leading to neuronal dysfunction. In this study, we aimed to investigate the lysosomal changes in CLN5-deficient human neurons. We used an induced pluripotent stem cell system, which generates pure human cortical-like glutamatergic neurons. Using CRISPRi, we inhibited the expression of CLN5 in human neurons. The CLN5-deficient human neurons showed reduced acidic organelles and reduced lysosomal enzyme activity measured by microscopy and flow cytometry. Furthermore, the CLN5-deficient human neurons also showed impaired lysosomal movement—a phenotype that has never been reported in *CLN5* Batten disease. Lysosomal trafficking is key to maintain local degradation of cellular wastes, especially in long neuronal projections, and our results from the human neuronal model present a key finding to understand the underlying lysosomal pathology in neurodegenerative diseases.

## 1. Introduction

Batten disease or neuronal ceroid lipofuscinosis (NCL) is a group of rare, fatal inherited neurological diseases predominantly affecting children and is characterised by symptoms including motor deficits, mental retardation, cognitive impairment, visual impairment, and epileptic seizures (reviewed in [1]). There are thirteen forms of Batten disease caused by mutations in thirteen *CLN* genes (*CLN1-8* and *10–14*) [2,3]. Mutations in one of the *CLN* genes, *CLN5*, cause a variant late-infantile NCL [4,5,6]. Life expectancy for children with *CLN5* mutations is between ten and thirty years, and there is currently no cure or approved treatment for *CLN5* Batten disease [1,4,6,7].

A cellular hallmark of Batten disease is defective lysosomes, the cellular waste recycling machinery [3]. Lysosomes are acidic vesicles containing hydrolytic enzymes, and loss of the lysosomal protein CLN5 shows reduced acidic organelles and impaired autophagy, as observed in ovine neural cultures from the naturally occurring CLN5-deficient sheep model [8]. Loss of CLN5 is further associated with different neuronal and non-neuronal pathologies, including accumulation of autofluorescent storage materials [4,9,10,11], impaired retromer trafficking and endosomal sorting [12,13,14], disturbed lipid metabolism [15], metal imbalance [16], and mitochondrial dysfunction [17]. A study in Dictyostelium found that Cln5 acts as a glycoside hydrolase [18] and the human CLN5 has been predicted to be a lysosomal protease [19]. However, a functional assay with purified human CLN5 has never been performed; hence, the protein function is still unknown. CLN5 interacts with other CLN proteins associated with Batten disease to maintain lysosomal homeostasis (reviewed in [1]).

To understand what other properties of the lysosomes are impaired due to the loss of CLN5, which leads to neuronal dysfunction, we inhibited CLN5 expression in human neurons. In this study, we developed a unique CLN5-deficient human neuronal model with isogenic controls from induced pluripotent stem cells (iPSCs) using clustered regularly interspaced short palindromic repeat interference (CRISPRi). In our CLN5-deficient human neuronal model, we confirm the reduced acidic organelles, as observed in our previous ovine study [8]. Intriguingly, we report, for the first time, that CLN5 deficiency in human neurons shows impaired lysosomal enzyme activity and impaired lysosomal movement, both being crucial for lysosomal homeostasis and, hence, maintaining neuronal health.

## 2. Materials and Methods

### 2.1. Generation of Induced Pluripotent Stem-Cell-Derived Human Neurons

**Figure 1:**
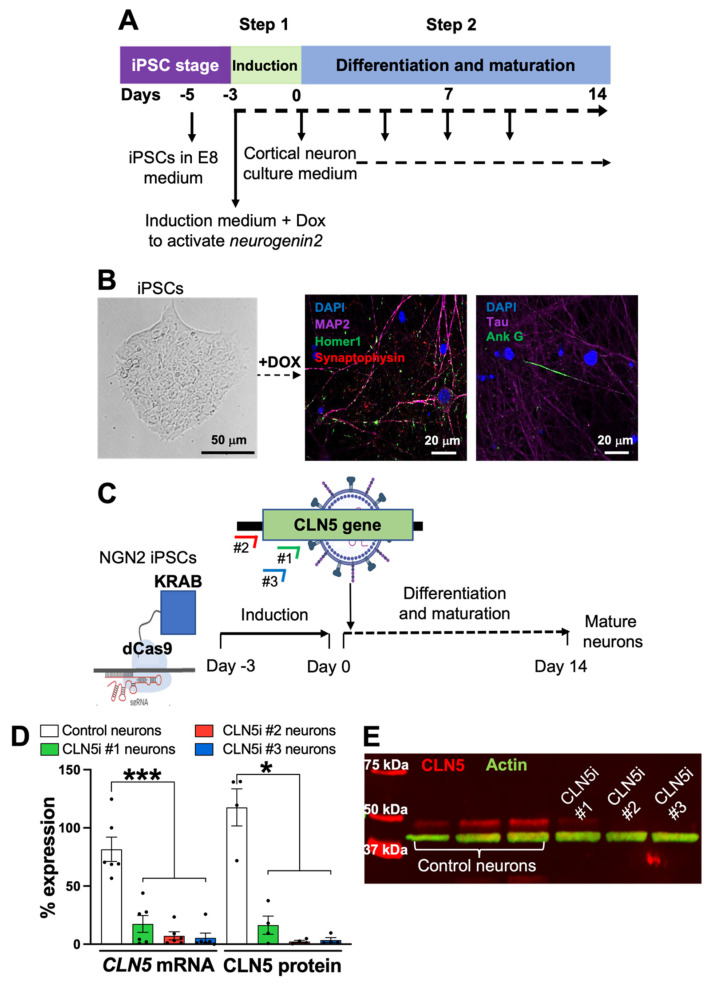
iPSC-derived human cortical-like glutamatergic neurons mimicking CLN5 Batten disease. (**A**) Schematic of iPSC-derived human neuron generation. Three days of induction and fourteen days of differentiation and maturation generates pure human cortical-like glutamatergic neurons. (**B**) iPSCs with integrated *neurogenin-2* can be efficiently differentiated into pure human cortical-like glutamatergic neurons expressing neuronal markers. (**C**) CRISPRi strategy to inhibit *CLN5* in iPSC-derived human neurons. Three sgRNAs against *CLN5* were transduced using lentiviruses into iPSC-derived human neurons containing dCas9 machinery. (**D,E**) All three sgRNAs showed significant inhibition of CLN5, both at the transcript (D) and protein levels (D,E). (E) First three lanes are control neurons, and following three lanes are CLN5i neurons. *n* ≥ 4, * *p* < 0.05, *** *p* < 0.001.

For this study, we used a published protocol established by Dr. Michael Ward’s group [20] to generate pure human cortical-like glutamatergic neurons from iPSCs. In short, the transcription factor *neurogenin-2* in a doxycycline-inducible cassette was stably integrated into the AAVS1 safe-harbour locus of iPSCs cultured in Essential 8 media (ThermoFisher Scientific, Auckland, New Zealand). The expression of *neurogenin-2* in the iPSCs was induced for three days using doxycycline (Sigma-Aldrich, Castle Hill, Australia). Post-induction, the differentiation of the *neurogenin-2* iPSCs into mature isogenic, integrated, and inducible pure human cortical-like glutamatergic neurons (i^3^Ns) was completed in two weeks in BrainPhys media (STEMCELL Technologies, Vancouver BC, Canada) (Figure 1A). After two weeks, the iPSC-derived mature neurons were fixed with 4% paraformaldehyde (Sigma-Aldrich, Castle Hill, Australia). The neurons were recognised using antibodies for neuronal markers (listed in Supplementary Tables) and stained with 4′,6-diamidino-2-phenylindole (DAPI) (Sigma-Aldrich, Castle Hill, Australia), as previously described [8]. The stained neurons were imaged (Figure 1B) on a Nikon A1+ Inverted Confocal Laser Scanning microscope (Nikon, Tochigi, Japan).

### 2.2. CRISPR Interference in iPSC-Derived Human Neurons

*Neurogenin-2* iPSCs with deactivated Cas9 (dCas9-KRAB) [21], tagged with a blue fluorescent protein (BFP) and stably integrated into the CLYBL safe-harbour locus [20], were induced to generate i^3^Ns, which have been used for the following experiments from at least three different passage numbers. To inhibit the expression of *CLN5*, three individual single-guide RNAs (sgRNAs) were designed against *CLN5*—two in the coding region (#1 and #3) and one in *CLN5* 5′UTR (#2) (Figure 1C), following Horlbeck et al. [22]. A control sgRNA was designed against a green fluorescent protein [23]. Following previously established protocols [2], the control and *CLN5* sgRNAs were cloned into Addgene plasmid #60955 (pU6-sgRNA EF1Alpha-puro-T2A-BFP) and then packaged into lentiviruses (Addgene plasmid #12259 and 12260). To inhibit *CLN5* in dCas9-i^3^Ns (henceforth termed as CLN5i neurons), induced dCas9-i^3^Ns were transduced with lentiviruses with either *CLN5* sgRNAs or control sgRNA on Day 1 of differentiation/maturation (Figure 1C) at a multiplicity of infection (MOI) of 1. The lentivirally transduced neurons were maintained until Day 14 with half-media changes on Days 4, 7, and 10 (Figure 1A). The Day 14 control and CLN5i neurons were harvested with Accutase (ThermoFisher Scientific, Auckland, New Zealand) to isolate total RNA using PureLink RNA isolation kit (ThermoFisher Scientific, Auckland, New Zealand), following the manufacturer’s instructions. The RNA isolates were treated with DNAseI (ThermoFisher Scientific, Auckland, New Zealand) and quantified using a Nanodrop spectrophotometer (ThermoFisher Scientific, ND-2000, Auckland, New Zealand), following our previously established protocol [8]. *CLN5* transcripts were measured from the control and CLN5i neuron RNA by quantitative polymerase chain reaction [24] using an iTaq Universal Probes one-step kit (Bio-Rad, Auckland, New Zealand) on a Roche 96-well thermocycler (Roche, Auckland, New Zealand), following the manufacturer’s instructions. Similarly, Day 14 control and CLN5i neurons were used to isolate total cell lysates using N-PER Neuronal Protein Extraction Reagent (ThermoFisher Scientific, Auckland, New Zealand), following the manufacturer’s instructions. The protein isolates were quantified and tested for CLN5 protein expression using Western blot analysis, as previously described [25]. *GAPDH* [24] and ACTIN were used for normalisation for the qPCR and Western blot analyses, respectively. Details of the qPCR probes and antibodies used are listed in Supplementary Tables.

### 2.3. Assessment of Lysosomal Function and Movement

To test whether there is a difference in lysosomal function in control and CLN5i neurons, LysoTracker Red DND 99 dye (ThermoFisher Scientific, Auckland, New Zealand), which stains acidic organelles, was used following our previously published protocol [8]. Induced control and CLN5i neurons were cultured for two weeks in BrainPhys media on a 96-well clear bottom plate (Corning, New York, NY, USA) (plated at 10,000 neurons/well for high-throughput image analysis), on a Nunc Lab-Tek II Chamber Coverglass (ThermoFisher Scientific, Auckland, New Zealand) (plated at 30,000 neurons/well for confocal microscopy) and on a 24-well cell culture plate (Corning, New York, NY, USA) (plated at 100,000 neurons/well for flow cytometry). The Day 14 mature neurons were stained with the LysoTracker Red dye following Best et al. [8]. The LysoTracker Red-stained control and CLN5i neurons were used for obtaining still images as well as videos using an Olympus FV3000 confocal microscope (Olympus, Tokyo, Japan). For high-content image analysis, the LysoTracker Red-stained neurons were further stained with Hoechst stain for 10 min at 37 °C; maintained in Phenol Red-free BrainPhys media (STEMCELL Technologies, 05791, Vancouver, BC, Canada); imaged on a Cytation 5 cell-imaging multi-mode reader (BioTek, Vermont, VT, USA); and analysed using Fiji/ImageJ (NIH, Bethesda, MD, USA) [26]. Each sample (control neurons and CLN5-deficient neurons) was assayed 3 times, with 3 technical replicates each time, and fluorescence intensity was measured from 4 images per replicate consisting of ≥100 neurons. For the flow cytometric analysis, neurons were stained as above followed by incubation with Accutase at 37 °C for 5 min. After Accutase incubation, neurons were harvested and resuspended in Phenol Red-free BrainPhys media followed by recording LysoTracker Red mean fluorescence intensity on a BD Fortessa (BD Biosciences, Franklin Lakes, NJ, USA). A total of 10,000 events were analysed for each sample.

To test the change in the lysosomal mass, we transduced the control and CLN5i neurons with a lentivirus-expressing, lysosome-associated membrane protein 1 (LAMP1), tagged with neonGreen that would target all lysosomes and early endosomes. The neonGreen-tagged organelles in control and CLN5i neurons were quantified using flow cytometry, as described above.

For assessing cathepsin B activity (Magic Red assay kit, Abcam, Burlingame, KS, USA) in the control and CLN5i neurons, Magic Red dye was diluted in the ratios of 1:150 and 1:250 for high-content imaging and confocal imaging, respectively, in Phenol Red-free BrainPhys media. The control and CLN5i neurons were incubated with the diluted Magic Red dye for 30 min at 37 °C followed by staining with a Hoechst stain for 10 min at 37 °C. The stained neurons were maintained in Phenol Red-free BrainPhys media for imaging as described earlier.

For the lysosomal movement analysis, time-lapse videos were obtained on an Olympus FV3000 confocal microscope (Olympus, Tokyo, Japan). For one video, 60 images were taken at 1 frame per 5 s for 5 min. At least two videos were obtained per sample, with ten kymographs generated per video, and the experiments were performed in three independent replicates. The resulting videos were analysed using Kymograph Clear [27] to generate ten kymographs per video. The kymographs were further analysed for lysosomal movement using KymoButler [28].

### 2.4. Statistical Analysis

All experiments were performed *n* ≥ 3 times and analysed on GraphPad Prism (GraphPad, San Diego, CA, USA). For RNA, protein, LysoTracker Red microscopic and flow cytometry analyses, and Magic Red microscopic analysis, one-way ANOVA was used to assess statistical significance. For the lysosomal movement analyses, in addition to one-way ANOVA, we performed a two-way ANOVA analysis to assess the overall change in lysosomal movement in CLN5i neurons compared to control neurons. Data are presented as mean ± standard error of mean (SEM). * *p* < 0.05, ** *p* < 0.01, *** *p* < 0.001.

## 3. Results

### 3.1. Inhibition of CLN5 in Human Neurons

In our iPSC-derived neurons (Figure 1A), we confirmed the expression of neuronal markers (Figure 1B)—MAP2 for dendrites, Tau for axons, synaptophysin for pre-synaptic vesicles, Homer1 for postsynaptic structures, and ankyrin G for axon initial segment. The CRISPRi strategy adapted to inhibit the expression of *CLN5* in neurons gave more than 90% CLN5 inhibition for two sgRNAs (CLN5i #2 and #3 neurons) compared to the control, both at the transcript level (Figure 1D) and at the protein level (Figure 1D,E). The expression of *CLN5* in CLN5i #1 neurons was more than CLN5i #2 and #3 neurons, albeit lower than the control neurons. Hence, we continued using all three sgRNAs for the following analyses, in which CLN5i #2 and #3 neurons represent homozygous CLN5 knockout neurons and CLN5i #1 neurons represent heterozygous CLN5 neurons. Our CLN5-deficient neurons are the first human neuronal model with isogenic controls via which the associated neuronal pathogenesis can be studied.

### 3.2. Loss of CLN5 Reduces Acidic Organelles and Lysosomal Enzyme Activity in Human Neurons

**Figure 2:**
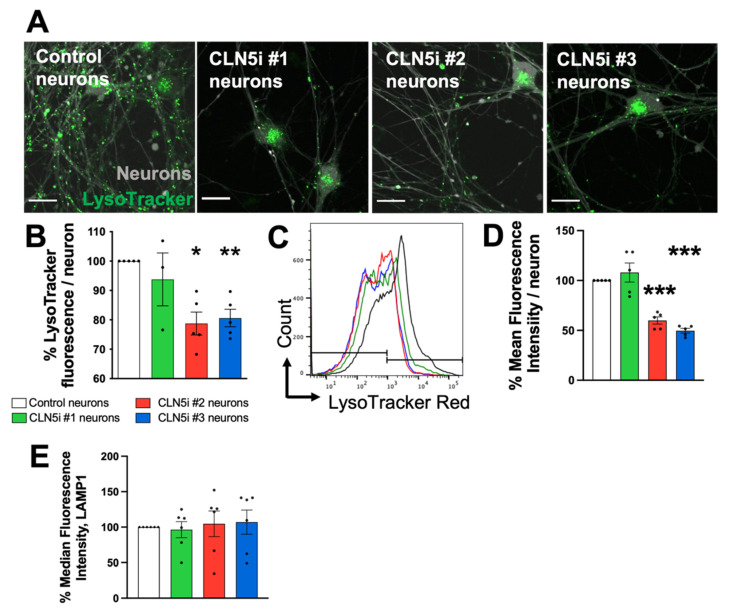
CLN5-deficient human neurons exhibit reduced acidic organelles. (**A,B**) Microscopic analysis of acidic organelles in the iPSC-derived human neurons revealed reduced acidic organelles (measured by LysoTracker—compare pseudo-colour green) in CLN5i neurons (#2 and #3) compared to control neurons. (**C,D**) The microscopic analysis of acidic organelles was validated by flow cytometric analysis. (**E**) The CLN5i human neurons did not show any difference in lysosome mass, measured by quantification of LAMP1-neuonGreen-positive organelles using flow cytometry. *n* ≥ 3, * *p* < 0.05, ** *p* < 0.01, *** *p* < 0.001. Scale bar = 20 µm.

As in our previously published experiments in CLN5 ovine cells [8], we observed reduced staining of acidic organelles by LysoTracker, without a change in total lysosomal mass in the CLN5-deficient human neuronal model (Figure 2A–E). LysoTracker Red stains acidic organelles, and the CLN5i #2 and #3 neurons showed a significant decrease in the LysoTracker signal compared to control neurons (Figure 2A,B). We verified our LysoTracker microscopy results by flow cytometric analyses, which supported the decrease in the LysoTracker signal in CLN5i neurons compared to control neurons (Figure 2C,D). The total lysosomal mass, measured by quantifying LAMP1-neonGreen-positive organelles, did not show any significant difference between control and CLN5i neurons (Figure 2E). Our result indicates that the lysosomal mass in the neurons are not altered by inhibiting CLN5; however, we observed a decrease in acidic organelles when CLN5 is inhibited.

**Figure 3:**
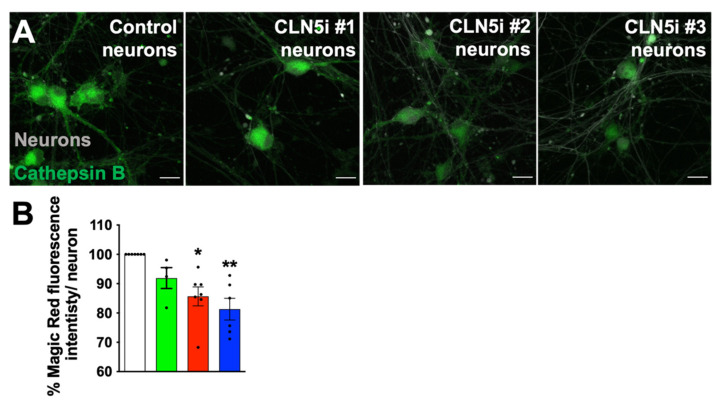
CLN5-deficient human neurons exhibit reduced lysosomal enzyme activity. (**A,B**) Microscopic analysis of lysosomal cathepsin B activity in the iPSC-derived human neurons revealed reduced enzyme activity (measured by Magic Red assay—compare pseudo-colour green) in CLN5-deficient neurons (#2 and #3) compared to control neurons. *n* ≥ 3, * *p* < 0.05, ** *p* < 0.01. Scale bar = 20 µm.

As in the case of the acidic organelle phenotype, the Magic-Red-stained control and CLN5i Day 14 neurons (Figure 3A,B) showed a decrease in cathepsin B activity. Our results suggest that the reduction in acidic organelles (lysosomes) in CLN5-deficient human neurons is perhaps leading to reduced activity of the lysosomal cathepsin B in the CLN5i neurons compared to the control neurons.

### 3.3. Loss of CLN5 Impairs Lysosomal Movement in Human Neurons

**Figure 4:**
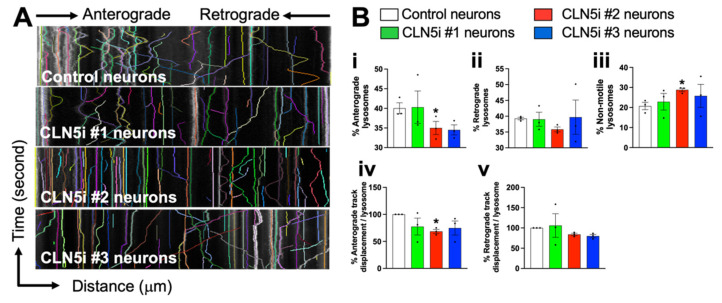
CLN5-deficient human neurons exhibit impaired lysosomal movement. (**A,B**) CLN5-deficient neurons (CLN5i #2 neurons) showed reduced percentage of anterograde lysosomes, increased percentage of non-motile neurons, and reduced anterograde track displacement compared to control neurons measured by confocal microscopy and kymograph analysis. *n* = 3, * *p* < 0.05.

Lysosomes travel to distal ends of neurons to maintain local degradative activities, therefore making lysosomal movement crucial to maintain homeostasis in the neurons [29]. Some CLN proteins have been recently shown to alter lysosomal positioning [14,30]; however, whether inhibiting CLN5 changes the lysosomal movement/trafficking in human neurons is unknown. To assess the lysosomal movement in control vs. CLN5i neurons, kymographs were generated from time-lapse videos of LysoTracker Red stained lysosomes in control vs. CLN5i neurons (Figure 4A). The generated kymographs show three features: (1) curved lines going from left to right represent lysosomes moving in the anterograde direction (i.e., away from the cell body); (2) curved lines going from right to left represent lysosomes moving in the retrograde direction (i.e., towards the cell body); and (3) straight lines represent non-motile lysosomes. The CLN5i neurons, especially CLN5i #2 neurons (Figure 4Bi), showed a significantly reduced percentage of anterograde lysosomes, whereas CLN5i #3 neurons showed a similar trend (Figure 4Bi). No statistically significant difference was observed for the percentage of retrograde lysosomes, although CLN5i #2 neurons showed a decreasing trend compared to the control neurons (Figure 4Bii). CLN5i neurons showed an increased percentage of non-motile lysosomes compared to the control neurons, with CLN5i #2 neurons showing a significant increase (Figure 4Biii). The direction of travel of the lysosomes—anterograde, retrograde, or non-motile—is a dependable variable for lysosomal movement. To test whether the overall movement of lysosomes was impaired in CLN5i vs. control neurons, a two-way ANOVA test was performed that showed a significant difference in lysosomal movement between CLN5i vs. control neurons (*p* < 0.01). When we tested the distance travelled by the lysosomes in control and CLN5i neurons, we observed that CLN5i #2 neurons showed a significant reduction in distance covered by the anterograde lysosomes (Figure 4Biv). CLN5i #2 and #3 neurons showed a decreasing trend in distance covered by the retrograde lysosomes compared to the control neurons; however, no statistical significance was achieved (Figure 4Bv).

Interestingly, we also observed some lysosomes that were motile but could not decide on the movement direction. As a result, these bidirectionally motile lysosomes kept moving back and forth without actually moving in their designated direction. This phenotype was prominent in CLN5i #2 and #3 neurons (Video S1B,C, encircled lysosomes) as compared to control neurons (Video S1A), which showed smooth uni-directional movement of the lysosomes.

## 4. Discussion

Our study, involving a unique human neuronal model mimicking CLN5 Batten disease with isogenic controls, revealed (1) our capability to model Batten disease in a dish using CRISPRi and iPSC-derived human neurons; (2) that >90% inhibition of CLN5 expression was achieved using CRISPRi in iPSC-derived human neurons; and that CLN5-deficient human neurons manifest defects in (3) acidic organelles (lysosomes), (4) lysosomal enzyme activity, and (5) lysosomal movement. Our study is the first to report that CLN5 deficiency in human neurons can lead to impaired lysosomal function and lysosomal movement.

Lysosomes are acidic organelles, and lysosomal acidity is crucial to maintain a working environment for lysosomal degradative enzymes. Hence, the reduction in the acidity of organelles in CLN5-deficient neurons (Figure 2A–D) would render the lysosomal enzymes inactive (Figure 3A,B), which, in turn, would impair degradation of cellular waste, ultimately leading to toxic build-up of cellular waste and an increase in neurotoxicity. Johnson et al. showed that peripheral lysosomes are less acidic than juxtanuclear ones and have reduced expression of Rab7 [31]. An effector of RAB7A, RILP, regulates the activity of V-ATPase, the key component for maintaining the acidity in the lysosomal lumen [32]. Interestingly, loss of CLN5 leads to reduced RAB7A/RILP interaction [14], which could lead to even further reduction in acidity in peripheral lysosomes, as seen in Figure 2A (second, third, and fourth panels).

Lysosomes travel throughout neurons, including axons and dendrites, as lysosomal transport is crucial not only for waste clearance in neuronal projections but also for lysosomal secondary neuronal functions, such as synapse regulation and cargo transport [33,34,35,36]. Retrograde transport allows lysosomes to fuse with autophagosomes near the cell body in neurons [37], whereas anterograde lysosomes are known to carry out degradative activities at the distal ends of axons [29]. In our in vitro human neuronal cultures, to include both axonal and dendritic functional anterograde and retrograde lysosomes, we imaged the LysoTracker-positive acidic lysosomes to understand the effect of CLN5 inhibition on lysosomal movement in the whole neuron. From our data, it is apparent that anterograde lysosomal movement is impaired in CLN5-deficient human neurons, whereas retrograde lysosomal movement shows a trend of impairment (not statistically significant). Dysfunctional lysosomal trafficking could result in the failure of lysosomes to fuse with autophagosomes, leading to an impaired autophagy–lysosomal pathway, as seen in our previous study [8]. Impaired lysosomal function in neurodegenerative diseases is not uncommon [38,39]. Hence, it will be interesting to test whether other forms of Batten disease as well as other neurodegenerative diseases show such lysosomal trafficking anomalies. Furthermore, testing whether the expression or regulation of lysosome trafficking machinery is altered in Batten disease might reveal novel regulatory defects in the affected neurons.

In the study by Uusi-Rauva et al. (2017) [40], the authors reprogrammed a skin fibroblast from a patient with CLN5 Batten disease to generate iPSCs and differentiated the iPSCs into neural cells. In the iPSC-derived neural cells with *CLN5* mutation, the authors revealed that LAMP1-stained organelles showed an increased area and intensity. Although we did not measure lysosomal area in our iPSC-derived CLN5-deficient human neurons, we noticed an increased number of non-motile lysosomes (Figure 4B) and an increase in lysosomal intensity in the cell body (Figure 2A: second, third, and fourth panels) of CLN5-deficient neurons, with the overall lysosomal mass remaining the same (Figure 2E). The increased number of non-motile lysosomes and impaired anterograde lysosomal movement explain the accumulation of lysosomes in the cell bodies of the CLN5-deficient neurons (Figure 2A: second, third, and fourth panels, and Video S1B,C), as reported by Uusi-Rauva et al. Yasa et al. (2021) [14] suggested that CLN5^KO^ HeLa cells were not able to move lysosomes as efficiently as wild-type HeLa cells. In our study, the impaired anterograde movement in the CLN5-deficient human neurons could indicate that the neuronal projections are being exempt from having their waste cleared, which could cause neuronal toxicity at the synapses. We also tested velocity of the lysosomes in the control and CLN5-deficient neurons, with no significant differences observed in anterograde or retrograde lysosomal velocity (Figure S1A,B).

Overall, our iPSC-derived human neurons offer a wide range of applications from CRISPR manipulation to imaging specific organelle localisation and movement. Our human neuronal system offers an unprecedented platform for mimicking all forms of Batten disease in human neurons to study the interactions of the thirteen *CLN* genes in the development and progression of the disease, to test neuronal phenotypes, and to screen therapeutic candidates for Batten disease.

## Data Availability

Data is contained within the article or Supplementary Material.

## Supplementary Materials

The following are available online at https://www.mdpi.com/article/10.3390/biom11101412/s1. Table S1: Primary antibodies used. Table S2: Secondary antibodies used. Table S3: qPCR probes used. Figure S1: CLN5-deficient human neurons did not show a difference in the velocity of the motile lysosomes. CLN5-deficient neurons did not show any difference in the anterograde (A) and retrograde (B) velocity of lysosomes compared to the control neurons. The velocities of the lysosomes were measured using kymograph analysis as described in Section 2.3. Figure S2: Full Western blot image of lysates from control and CLN5i human neurons probed with anti-CLN5 and anti-actin antibodies. Video S1: CLN5-deficient human neurons showed aberrant lysosomal movement. Time lapse videos of acidic lysosomes were obtained for 5 min on a confocal microscope. CLN5i neurons (B,C) showed aberrant movement of the lysosomes (see encircled lysosomes); i.e., some lysosomes seem to be lacking a uni-directional movement and kept moving back and forth without actually moving in their designated direction, as compared to the smooth uni-directionally moving lysosomes in control neurons (A). Scale bar = 20 µm.
