# Supplementary material for: Deficiency of the Lysosomal Protein CLN5 Alters Lysosomal Function and Movement
Source: Biomolecules. 2021 Sep 27;11(10):1412. doi: 10.3390/biom11101412 (PMC8533494; doi:10.3390/biom11101412)
Supplement: Supplementary file 1 [file biomolecules-11-01412-s001.zip › SM/Supplementary_Tables.pdf]

Table S1: Primary antibodies used

Target protein	Species	Mono/polyclonal	Dilution used	Vendor	Catalogue no.
MAP2	Guinea pig	Polyclonal	1:2000	Synaptic systems	188004
HOMER 1B/C	Rabbit	Polyclonal	1:1000	Synaptic systems	160022
SYNAPTOPHYSIN	Mouse	Monoclonal	1:1000	Synaptic systems	101011
TAU	Guinea pig	Polyclonal	1:2000	Synaptic systems	314004
ANKYRIN G	Mouse	Monoclonal	1:200	Invitrogen	338800
CLN5	Rabbit	Monoclonal	1:1000	Abcam	170899
ACTIN	Mouse	Monoclonal	1:2000	Novus Biologicals	NB100-74340

Table S2: Secondary antibodies used

Target protein	Species	Dilution used	Vendor	Catalogue no.
Anti Guinea Pig IgG (H+L) Highly Cross-Adsorbed Secondary Antibody, Alexa Fluor 647	Goat	1:1000	Invitrogen	A-21450
Anti-Rabbit IgG (H+L) Highly Cross-Adsorbed Secondary Antibody, Alexa Fluor 488	Goat	1:1000	Invitrogen	A11034
Anti-Mouse IgG (H+L) Highly Cross-Adsorbed Secondary Antibody, Alexa Fluor 594	Goat	1:1000	Invitrogen	A11032
Anti-Mouse IgG (H+L) Highly Cross-Adsorbed Secondary Antibody, Alexa Fluor 488	Goat	1:1000	Invitrogen	A11029
IRDye® 680RD Anti-Mouse IgG Secondary Antibody	Goat	1:20000	Licor	926-68070
IRDye® 800RD Anti-Rabbit IgG Secondary Antibody	Goat	1:20000	Licor	926-32211

Table S3: qPCR probes used

Target protein	Unique assay ID	Dilution used	Vendor	Catalogue no.
CLN5	qHsaCEP0050205	1:20	Bio-Rad	10031228
GAPDH	qHsaCEP0041396	1:20	Bio-Rad	10031226
