# Supplementary figures and images for: Deficiency of the Lysosomal Protein CLN5 Alters Lysosomal Function and Movement
Source: Biomolecules. 2021 Sep 27;11(10):1412. doi: 10.3390/biom11101412 (PMC8533494; doi:10.3390/biom11101412)

# Figure S1

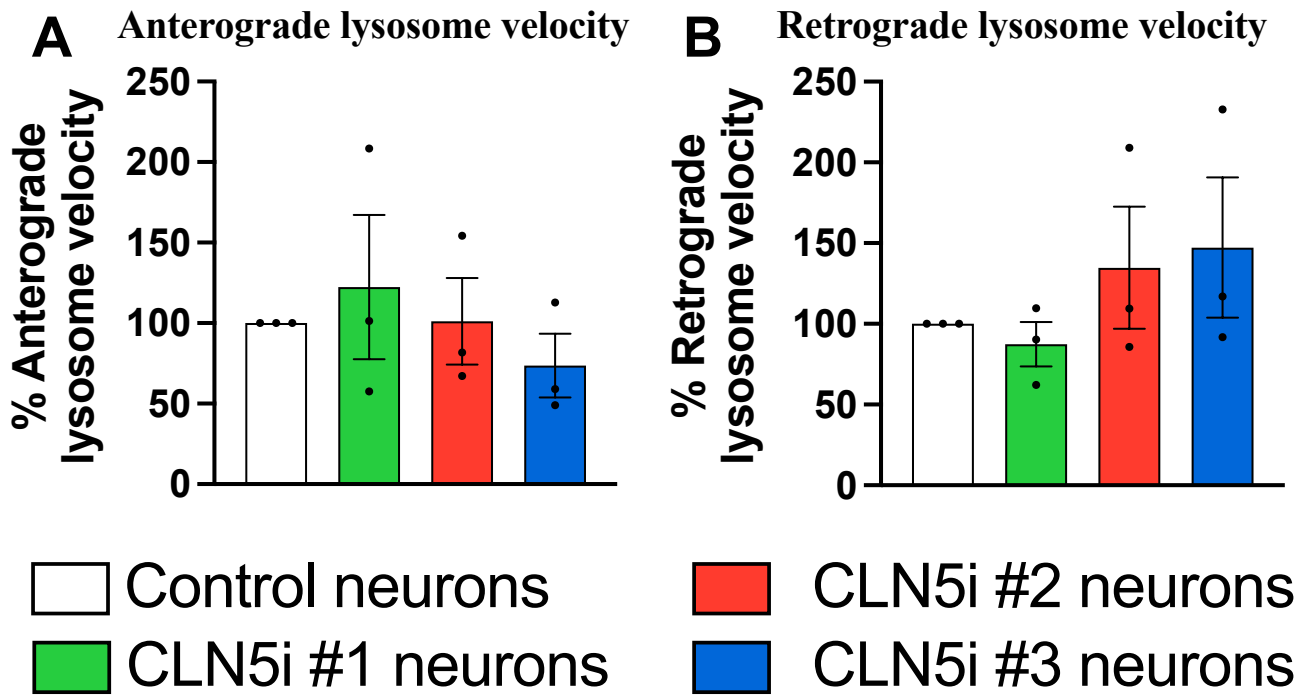

# Figure S2

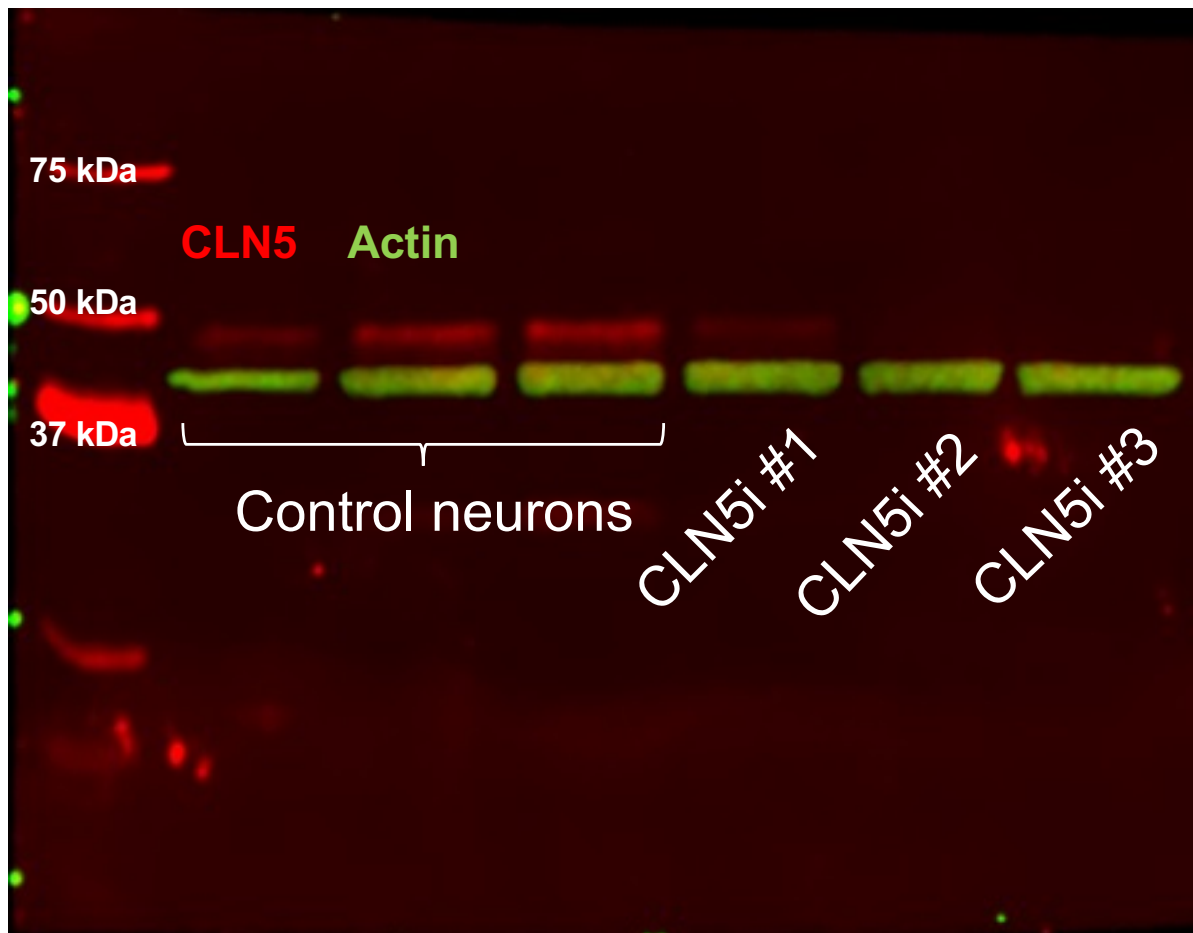

Supplement: Supplementary file 1 [file biomolecules-11-01412-s001.zip › SM/Supplementary_Figures.pdf]
